# Determinants of self-care among Jordanian children with type 1 diabetes mellitus

**DOI:** 10.1186/s42506-024-00166-8

**Published:** 2024-08-20

**Authors:** Salam Hamdan, Esra’ Taybeh, Mervat M. Alsous

**Affiliations:** 1 Faculty of Pharmacy, Isra University, Amman, Jordan; 2 Faculty of Pharmacy, Yarmouk University, Irbid, Jordan

**Keywords:** Type 1 diabetes mellitus, Self-care, Knowledge, Psychological well-being

## Abstract

**Background:**

Diabetes Self-Care Management (DSCM) is crucial for managing diabetes mellitus and improving patients’ well-being. Research on the young age group in Jordan is limited, and there is a lack of studies using an evaluation tool for understanding diabetes pharmacotherapy. This study intends to fill the information gap by examining young Jordanian patients’ knowledge and comprehension of type 1 diabetes mellitus (T1DM) and its treatment modalities, evaluating their psychological well-being, and examining the relationship between children’s psychological health and self-care.

**Methods:**

This cross-sectional study was conducted in the Jordanian Ministry of Health hospitals in Amman from June 2021 to January 2022. A convenience sampling method was used to select Arabic-speaking diabetic patients aged 11-a8 years who provided signed consent. A sample size of 400 was estimated. A self-administered questionnaire was developed based on a literature review to assess sociodemographic characteristics and diabetes and insulin knowledge, and validated scales were used to assess self-management (SMOD-A) and psychological well-being (ChilD-S).

**Results:**

Analysis of the questionnaire responses revealed varying levels of knowledge among the participants. Approximately half of the children (49.0%) demonstrated a lack of knowledge of diabetes pharmacotherapy. Psychological well-being indicators indicated moderate levels of happiness and feeling fine. The analysis of self-management indicators highlighted areas for improvement. Positive weak but significant correlations were found between children’s knowledge about diabetes (*r* = 0.255, *p* < 0.01), diabetes pharmacotherapy knowledge (*r* = 0.125, *p* < 0.05), psychological well-being (*r* = 0.112, *p* < 0.05), and their diabetic self-management scores. A multivariate regression analysis identified predictors of self-management, including the child’s school year (*p* = 0.035), ability to express feelings (*p* = 0.039), recent HbA1c levels (*p* = 0.028), and diabetes knowledge score (*p* < 0.001).

**Conclusion:**

Participants exhibited varying levels of knowledge about diabetes pharmacotherapy and self-management. Knowledge about diabetes was identified as a predictor for effective self-management. Moreover, glycemic control and diabetes mellitus awareness majorly impact overall self-management behaviors. Tailored education programs are necessary to fill knowledge gaps and enhance diabetes management among children.

## Introduction

Diabetes Self-Care Management (DSCM) plays a crucial role in the management of diabetes mellitus and is essential for patients’ overall well-being and quality of life [1–3]. DSCM involves various self-care practices such as diet, physical activity, blood glucose monitoring, and adherence to medication, which all help to maintain optimal glucose levels [4].

A strong correlation exists between consistent engagement in DSCM and better health outcomes, including proper blood glucose control, reduced complications, improved quality of life, and lower mortality rates [5, 6]. Despite the importance of DSCM, many type 1 diabetes mellitus (T1DM) patients struggle with adherence to self-care practices, and several enablers and barriers affect their decision-making approach to self-management [7]. Enablers such as knowledge and education about the disease, understanding the link between self-care practices and health outcomes, and adequate self-care management skills [8, 9] play a significant role in patients’ successful disease control. On the other hand, barriers such as depressive symptoms, diabetes-associated distress, difficulty in performing lifestyle modifications, lack of psychological support, fear of hypoglycemia, and poor communication with healthcare providers negatively impact self-care management [10–13].

Several studies have also reported that psychological and psychiatric disorders, including depression and fear of hypoglycemia, can significantly impact diabetes mellitus (DM) and its associated complications [14–17].

In light of the gap in knowledge on the young age group in Jordan, this study aims to explore the knowledge and understanding of T1DM and its treatment strategies among young Jordanian patients, assess their psychological well-being, and investigate the impact of child knowledge and psychological well-being on self-care. The study’s unique focus and multi-dimensional approach significantly contribute to the existing body of knowledge in diabetes research, potentially enhancing the quality of care and support provided to young individuals with T1DM in Jordan.

## Methods

### Study design and settings

A cross-sectional study was conducted in Jordanian Ministry of Health hospitals located in Amman, the Jordanian capital, over 8 months (from June 2021 to January 2022). These hospitals include Al Bashir Hospital, Al Zarqa Government Hospital, and Prince Faisal Governmental Hospital. The survey was administered in the waiting area of the endocrinology clinic within these hospital facilities. The approval to conduct the study was obtained from the ethics committee at the School of Pharmacy, Isra University, and the Jordan Ministry of Health. Written consent was obtained from the patients and their parents.

### Study population and sample size

A convenience sampling method was used to select the adolescents from the daily attendees who visit the selected Jordanian Ministry of Health hospitals. This type of sample was chosen for the ease of accessibility and availability of eligible participants. The researchers approached individuals who were readily available at the hospitals during the study period. The study population consisted of patients with diabetes aged 11–18 years, specifically those with type 1 diabetes, who were Arabic speakers and provided a signed consent form. The exclusion criteria included those with mental disorders and those who did not give consent to participate in the study. The sample size was estimated to detect the true proportion of children with poor self-management with 95% confidence and a margin of error equal to 5%. To account for maximum variability, the proportion was assumed to be 50%. The desired sample size was 384 children with diabetes, and the final sample size achieved was 400 children.

### Study tool

The study tool was designed by reviewing previous studies about the knowledge of adolescents with type 1 diabetes, self-care, and their psychological state [18–22]. The self-administered questionnaire was comprised of four major sections with a total of 81 questions; the first section (six questions) assesses the patients’ sociodemographic, past medical history, and results of clinical tests like the glycated hemoglobin (HbA1c) where the last reading was extracted from hospital records. Section two measures the patients’ knowledge of both the diabetes mellitus disease (DKQ) (24 questions), diabetes mellitus management (three questions) using true/false questions, and the focused pharmacotherapy knowledge assessment tool [18, 21]. The third Sect. (40 questions) measures the children’s perceived level of diabetic self-care via the Self-Management of Type 1 Diabetes in Adolescents scale (SMOD-A) [20]. The last Sect. (8 questions) measures the level of psychological well-being of children with diabetes via administering the Children’s Depression Screener (ChilD-S) scale [19].

An Arabic translation and a validation of the questionnaires were conducted. The questionnaires were translated from English to Arabic by qualified bilingual translators, to ensure accuracy and cultural equivalence. Four researchers and experts at the university and hospitals reviewed the translations for content validity and linguistic appropriateness. Pilot testing with ten healthy Arabic-speaking individuals provided feedback for necessary revisions, resulting in final versions suitable for the target population. The data from these healthy participants were not included in the final analysis. The survey was paper-based and filled out in the presence of the researcher. Respondents, on average, took between 20 and 30 min to complete the questionnaire.

### Study measures

#### (a) Diabetes Knowledge Questionnaire

The Diabetes Knowledge Questionnaire (DKQ) is a 24-item questionnaire [21] developed to assess individuals’ overall knowledge of diabetes based on the content guidelines outlined in the National Standards for Diabetes Patient Education Programs [23]. The DKQ, with a reported reliability coefficient of 0.78, serves as a reliable and valid measure of diabetes-related knowledge and can be administered with relative ease. Participants are provided with response choices of “Yes,” “No,” and “I don’t know.” Each item is scored as either correct or incorrect (selecting “I don’t know” is considered an incorrect response), and the sum of correct responses is calculated to attain a total score. The resulting knowledge score was bounded between 0 and 24 points.

#### (b) An evaluation tool for the understanding of pharmacotherapy

The patients’ understanding of pharmacotherapy was assessed based on the framework proposed by Ceccato et al. [22]. The evaluation involved asking questions (3 questions) that covered various aspects, including the drug’s name, dosage, and frequency of use of insulin. The researcher transcribed and interpreted the responses provided by the patients, comparing them with the information from medical prescriptions and records. In cases of disagreement, a second researcher was consulted. The researchers involved in this process hold at least a bachelor’s degree in pharmacy and possess clinical experience in a hospital.

The knowledge score of diabetes mellitus pharmacotherapy was computed based on the name, dosage, and frequency of insulin use, with each question scored by two points and a total knowledge score bounded between 0 and 6 points. The scoring system for the questionnaire was as follows: incorrect or unknown responses were given a score of 0 points, partial correct answers received a score of 1 point, and complete correct answers were rewarded with a score of 2 points. The maximum attainable score was 6 points, indicating correct responses for all items.

#### (c) Children’s Depression Screener (ChilD-S) (19)

Children’s Depression Screener (ChilD-S) Questionnaire is comprised of eight questions designed to assess the psychological well-being of children, specifically targeting child depression. This questionnaire employs a 5-point Likert scale (ranging from “Strongly Agree” to “Strongly Disagree”) to measure the responses of the patients [19].

The mean and standard deviation (SD) were calculated for the responses to reflect the average response across all subjects, capturing the overall trend and central tendency of the data. This strategy permits the identification of general patterns and variations within the sample, which might be overlooked while using a strict cut-off. The ChilD-S scale demonstrated substantial internal consistency, with a Cronbach’s alpha coefficient of 0.72.

#### (d) Self-Management of Type 1 Diabetes in Adolescents Scale (SMOD-A) (20)

The Self-Management of Type 1 Diabetes in Adolescents scale (SMOD-A) is a self-reported assessment tool designed to measure diabetes self-care skills and to aid healthcare providers in evaluating and promoting self-management in adolescents with type 1 diabetes [20]. The SMOD-A consists of 40 questions measuring five subscales of self-management, including Collaboration with Parents, Diabetes Care Activities, Diabetes Problem Solving, Diabetes Communication, and Goals. The original questionnaire consists of 52 questions; however, certain questions were omitted in our study, to ensure the measure’s suitability for the Jordanian culture. The adapted SMOD-A Questionnaire exhibited high internal consistency, as evidenced by a Cronbach’s alpha coefficient of 0.89.

The SMOD-A questionnaire was administered using 0–3 Likert-like scales (from Never to always) to measure the children’s perceived level of diabetic self-care. The mean and standard deviation (SD) were calculated for the responses to provide summary statistics.

### Statistical data analysis

Descriptive statistics including mean, standard deviations, frequencies, and percentages were used to describe the data. Internal consistency was evaluated using Cronbach’s alpha for Likert-like questionnaires and Kuder-Richardson’s (KR-20) test for binary questionnaires. Bivariate Pearson’s product-moment correlation was used to examine the correlation between continuous variables.

Multivariate linear regression analysis was used to examine the association between the SMOD-A Score, sociodemographic characteristics, knowledge of diabetes and treatment, and psychological well-being. The associations were expressed as multivariate-adjusted odds ratios (OR) with their associated 95% confidence intervals. The Statistical Package for the Social Sciences (SPSS) IBM statistical data analysis program version 20 was used, with the alpha significance level set at 0.05.

## Results

### Characteristics of the study’s sample

The number of children who participated in the study was 400, 60.8% were girls and 39.2% were boys. In terms of age distribution, 56.0% of the children belonged to the 11–15 years age group, while 44.0% were in the 16–18 years age group. Regarding the current school year, the highest representation was seen in the 12th grade (22.0%), followed by the 9th grade (15.5%). The mean HbA1c level of the children in the last month was 9.75% (SD = 2.56), with 80.3% having an HbA1c level of 8% or higher, while 19.8% had an HbA1c level below 8% (Table 1).

**Table 1:** The demographic characteristics of the sample of children with type 1 diabetes, Ministry of Health hospitals, Jordan, 2021–2022 (*n* = 400)

Characteristic	Classification	Frequency	Percentage (%)
Child’s gender	Girl	243	60.8
	Boy	157	39.2
Child’s age group	11–15 years	224	56.0
	16–18 years	176	44.0
Current school year	4th grade	75	18.8
	5th grade	32	8.0
	6th grade	37	9.2
	7th grade	29	7.2
	8th grade	30	7.5
	9th grade	62	15.5
	11th grade	47	11.8
	12th grade	88	22.0
Children’s ability to express feelings/anxiety verbally or non-verbally	No	189	47.2
	Yes	211	52.8
Glycated hemoglobin (Hba1c) Level	Hba1c < 8%	79	19.8
	Hba1c ≥ 8%	321	80.2
Last month’s glycated hemoglobin (Hba1c) level, mean (SD)	9.75 (2.56)		

### Children’s knowledge about diabetes mellitus

The results of the DKQ (Table 2) revealed varying levels of correct and incorrect answers among the participants. The main cause of diabetes, which is the lack of effective insulin, was correctly identified by 71.8% of the participants. Cuts and abrasions on diabetes heal more slowly was correctly answered by a similar proportion, while only 14.0% of the participants provided correct answers to the question that a person with diabetes should cleanse a cut with iodine and alcohol. The correct answer percentages ranged from 14.0 to 71.8%.

**Table 2:** Descriptive analysis of the children’s measured knowledge of diabetes (DKQ), Jordan, 2021–2024

Item	Correct answer #	Correctly answered (%)
Eating too much sugar and other sweet foods is a cause of diabetes	F	141 (35.3)
The usual cause of diabetes is a lack of effective insulin in the body	T	287 (71.8)
Diabetes is caused by the failure of the kidneys to keep sugar out of the urine	F	185 (46.3)
Kidneys produce insulin	F	253 (63.3)
In untreated diabetes, the amount of sugar in the blood usually increases	T	268 (67.0)
If I am diabetic, my children have a higher chance of being diabetic	T	232 (58.0)
Diabetes can be cured	F	193 (48.3)
A fasting blood sugar level of 210 is too high	T	226 (56.5)
The best way to check my diabetes is by testing my urine	F	214 (53.5)
Regular exercise will increase the need for insulin or other diabetic medication	F	230 (57.5)
There are two main types of diabetes: Type 1 (insulin-dependent) and Type 2 ( noninsulin-dependent)	T	262 (65.5)
An insulin reaction is caused by too much food	F	81 (20.3)
Medication is more important than diet and exercise to control my diabetes	F	224 (56.0)
Diabetes often causes poor circulation	T	177 (44.3)
Cuts and abrasions for diabetics heal more slowly	T	287 (71.8)
Diabetics should take extra care when cutting their toenails	T	280 (70.0)
A person with diabetes should cleanse a cut with iodine and alcohol	F	56 (14.0)
The way I prepare my food is as important as the foods I eat	T	236 (59.0)
Diabetes can damage my kidneys	T	248 (62.0)
Diabetes can cause loss of feeling in my hands, fingers, and feet	T	263 (65.8)
Shaking and sweating are signs of high blood sugar	F	210 (52.5)
Frequent urination and thirst are signs of low blood sugar	F	233 (58.3)
Tight elastic hose or socks are not bad for diabetics	F	214 (53.6)
A diabetic diet consists mostly of special foods	F	103 (25.8)

^**#**^The correct answer to each diabetic knowledge question is indicated by either T (True) or F (False) based on Garcia et al. [21]

### Children with diabetes knowledge of insulin

**Fig. 1:**
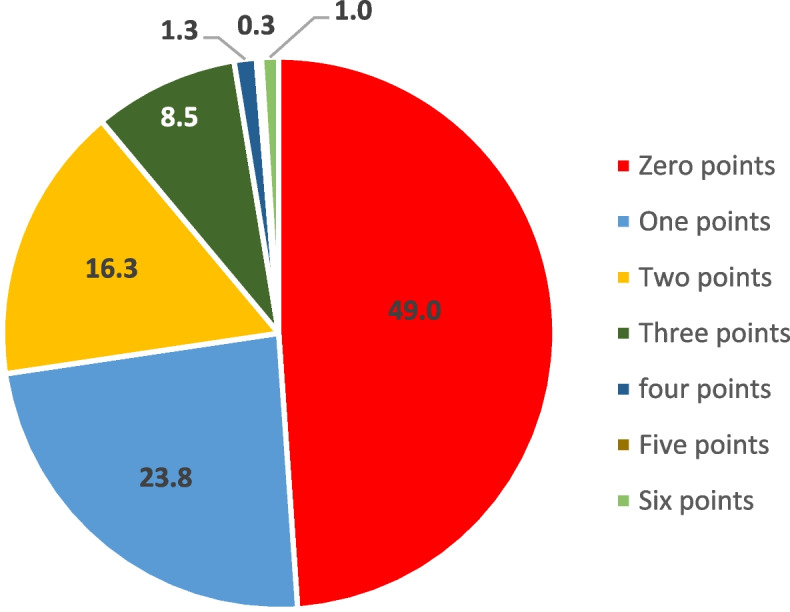
Knowledge scores (percentage) of children with diabetes about diabetes pharmacotherapy (mean diabetic pharmacotherapy knowledge score = 1, SD = 1.2)

Around half of the children (49%) scored zero points on the pharmacotherapy knowledge scale, indicating a lack of knowledge in this area. Around 40% scored one to two points, suggesting a limited understanding of diabetes pharmacotherapy. Notably, few children demonstrated a higher level of knowledge, with only a small proportion scoring three or more points on the pharmacotherapy knowledge scale (Fig. 1).

### Psychological well-being of children with diabetes

The analysis of the children’s psychological feelings of well-being revealed a mixed profile, with moderate levels of happiness and feeling fine (Table 3).

**Table 3:** Responses of Jordanian children with diabetes to the CHILD-S questionnaire

Feeling	Strongly agree *N* (%)	Agree *N* (%)	Neutral *N* (%)	Disagree *N* (%)	Strongly disagree *N* (%)	Mean	SD
I am happy	62 (15.5)	223 (55.8)	104 (26.0)	11 (2.8)	0 (0.0)	3.33	1.37
I am doing fine	179 (44.8)	52 (13.0)	77 (19.3)	59 (14.8)	33 (8.3)	3.71	1.38
I feel exhausted by everything	152 (38.0)	58 (14.5)	91 (22.8)	74 (18.5)	25 (6.3)	2.41	1.32
I worry a lot	162 (40.5)	71 (17.8)	71 (17.8)	71 (17.8)	25 (6.3)	2.32	1.33
I feel sad	156 (39.0)	66 (16.5)	86 (21.5)	68 (17.0)	24 (6.0)	2.35	1.31
I get upset quickly	157 (39.3)	108 27.0)	61 (15.3)	55 (13.8)	19 (4.8)	2.18	1.22
I am not in the mood for anything	147 (36.8)	78 (19.5)	80 (20.0)	65 (16.3)	30 (7.5)	2.38	1.32
I often think I did something wrong	143 (35.8)	55 (13.8)	106 (26.5)	64 (16.0)	32 (8.0)	2.47	1.33

### Children with diabetes self-management

The descriptive analysis of the subscales of the SMOD-A questionnaire revealed that the mean scores of parental collaboration and cooperation ranged from 2.14 to 2.83. For the child’s diabetic self-care activity subscale, the mean scores ranged from 1.85 to 2.68. The subscales ranged from 2.38 to 2.85 for problem-solving, 2.38 to 2.64 for communication, and 2.50 to 2.63 for goal setting (Table 4). The highest mean score was observed for the attention to readings (mean = 2.83, SD = 1.17), indicating relatively high levels of engagement in this aspect of self-care. On the other hand, the lowest self-rated diabetic self-care activities according to the children were abiding by carrying something saying that they are diabetic (like a bracelet) (mean = 1.85, SD = 0.94), suggesting potential challenges or lower adherence to this particular self-care activity.

**Table 4:** Children’s responses to the Diabetes Self-Management Scale (SMOD-A)

Subscale	Always (*n*, %)	Often (*n*, %)	Sometimes (*n*, %)	Never (*n*, %)	Mean	SD
**Child parental collaboration/cooperation subscale**						
Help with insulin decisions	103 (25.8)	44 (11.0)	124 (31.0)	129 (32.3)	2.30	1.17
Insulin telling	57 (14.2)	72 (18.0)	141 (35.3)	130 (32.5)	2.14	1.03
Calculating carbohydrates	66 (16.5)	86 (21.5)	114 (28.5)	134 (33.5)	2.21	1.08
Attention to readings	172 (43.0)	61 (15.3)	95 (23.8)	72 (18.0)	2.83	1.17
Check insulin (missing = 1)	148 (73.0)	64 (16.0)	111 (27.8)	76 (19.0)	2.71	1.15
Dealing with high sugars #	102 (25.5)	108 (27.0)	128 (32.0)	62 (15.5)	2.63	1.03
Make sure the meter is checked (missing = 2)	142 (35.5)	66 (16.5)	108 (27.0)	82 (20.5)	2.67	1.16
Questions to parents about carbohydrates (missing = 2)	88 (22.0)	82 (20.5)	112 (28.0)	116 (29.0)	2.36	1.12
Insulin tuning # (missing = 3)	82 (20.5)	102 (25.5)	115 (28.7)	98 (24.5)	2.42	1.07
**Child diabetic self-care activity subscale**						
Check sugar before eating (missing = 1)	113 (28.2)	111 (27.8)	110 (27.5)	65 (16.3)	2.68	1.05
Check before you’re reminded (missing = 1)	72 (18.0)	96 (24.0)	153 (38.3)	78 (19.5)	2.41	1.00
Follow the plan or count	85 (21.3)	75 (18.8)	120 (30.0)	120 (30.0)	2.31	1.11
Carry glucose or sugars (missing = 1)	124 (31.0)	73 (18.3)	97 (24.3)	105 (26.3)	2.54	1.18
Ketone test	43 (10.8)	64 (16.0)	102 (25.5)	191 (47.8)	1.90	1.03
Keep a record of numbers (missing = 1)	84 (21.0)	82 (20.5)	136 (34.0)	97 (24.3)	2.38	1.07
If the sugar is low, treat it and check later	99 (24.8)	72 (18.0)	137 (34.3)	92 (23.0)	2.45	1.10
Need to remind insulin #	93 (23.3)	113 (28.2)	121 (30.3)	73 (18.3)	2.56	1.04
Insulin skipping #	65 (16.3)	67 (16.8)	109 (27.3)	159 (39.8)	2.10	1.10
Carry something that says diabetes	33 (8.3)	51 (12.8)	138 (34.5)	178 (44.5)	1.85	0.94
I don’t like it when someone mentions #	77 (19.3)	56 (14.0)	117 (29.3)	150 (37.5)	2.15	1.13
**Child diabetic problem-solving subscale**						
Insulin decision (missing = 1)	106 (26.5)	79 (19.8)	113 (28.2)	101 (25.3)	2.48	1.14
To find out insulin, consider sugar and what was eaten (missing = 1)	111 (27.8)	91 (22.8)	118 (29.5)	79 (19.8)	2.59	1.09
Adjusting insulin depends on numbers (missing = 1)	146 (36.5)	75 (18.8)	118 (29.5)	60 (15.0)	2.77	1.10
When exercising, I change my eating or insulin	107 (26.8)	89 (22.3)	101 (25.3)	103 (25.8)	2.50	1.14
If the sugar is high, use insulin	122 (30.5)	102 (25.5)	91 (22.8)	85 (21.3)	2.65	1.12
Remember the HbA1c (A1c) value from the last visit	168 (42.0)	78 (19.5)	81 (20.3)	73 (18.3)	2.85	1.16
Find out what HbA1c (A1c) should be	134 (33.5)	77 (19.3)	100 (25.0)	77 (19.3)	2.69	1.14
**Diabetic child communication subscale**						
When you get upset with diabetes, talk about it (missing = 1)	137 (34.3)	66 (16.5)	110 (27.5)	86 (21.5)	2.64	1.16
If it bothers you, talk to your parents (missing = 2)	75 (18.8)	95 (23.8)	135 (33.8)	93 (23.3)	2.38	1.04
Think about what you’re saying to the nurse or the doctor (missing = 1)	91 (22.8)	106 (26.5)	105 (26.3)	97 (24.3)	2.48	1.09
Call a nurse or doctor when you can’t get sugars in range (missing = 1)	102 (25.5)	89 (22.3)	133 (33.3)	75 (18.8)	2.55	1.07
Stay informed	118 (29.5)	100 (25.0)	93 (23.3)	89 (22.3)	2.62	1.13
Review records with a nurse or doctor	103 (25.8)	104 (26.0)	117 (29.3)	76 (19.0)	2.58	1.07
Time alone with a nurse or doctor	102 (25.5)	89 (22.3)	108 (27.0)	101 (25.3)	2.48	1.13
Telling friends about diabetes (missing = 5)	102 (25.2)	71 (17.8)	120 (30.0)	102 (25.5)	2.43	1.13
**Diabetic child goal-setting subscale**						
I take care of myself	112 (28.0)	89 (22.3)	137 (34.3)	62 (15.5)	2.63	1.05
I take responsibility alone for my diabetes care	87 (21.8)	142 (35.5)	107 (26.8)	64 (16.0)	2.63	1.00
Try not to have problems in the future (missing = 1)	88 (22.0)	138 (34.5)	107 (26.8)	66 (16.5)	2.62	1.00
Do with friends	91 (22.8)	99 (24.8)	141 (35.3)	69 (17.3)	2.53	1.03
Understanding the cause of blood sugar numbers (missing = 3)	96 (24.0)	92 (23.0)	122 (30.5)	87 (21.8)	2.50	1.08

^#^Items with reversed scores

A positive weak but significant correlation was found between the children’s score for knowledge about DM and their diabetic self-management SMOD-A score (*r* = 0.255, *p* < 0.010). Similarly, a bivariate analysis revealed a positive and significant yet weak correlation between children’s diabetes pharmacotherapy knowledge score and their diabetic self-management score (*r* = 0.125, *p* < 0.050). Furthermore, a positive weak, yet significant correlation was observed between the children’s psychological well-being and their mean perceived diabetic self-management score (*r* = 0.112, *p* < 0.050), indicating that as their psychological well-being improved, their diabetic self-management tended to increase correspondingly.

### Determinants of self-management in children with diabetes

A multivariate linear regression analysis (Table 5) was conducted to examine the predictors of the children’s Self-Management of diabetes (SMOD-A) overall score. The results revealed that the school year of the child emerges as a significant negative predictor (*p* = 0.035), implying that being in a higher school year is negatively correlated with children’s self-management. Similarly, the ability to express feelings serves as a significant negative predictor (*p* = 0.039), indicating that a lower ability to express feelings corresponds to a higher self-management score. Conversely, the diabetes disease knowledge score stands out as a significant positive predictor (*p* < 0.001), suggesting that enhanced knowledge about diabetes is linked to a higher score of children’s self-management. Furthermore, the recent serum glycated hemoglobin HbA1c level acts as a significant negative predictor (*p* = 0.028), suggesting that lower levels of HbA1c are associated with higher levels of children’s diabetes self-management. However, the child’s age, sex, psychological well-being score, and knowledge of diabetes pharmacotherapy score were not predictors of the SMOD-A overall score.

**Table 5:** Multivariate linear regression analysis of the predictors of the children's self-management (SMOD-A) overall score

Variables	Standardized coefficients beta	95.0% correlation coefficient		*P*-value
		**Lower bound**	**Upper bound**	
(Constant)		10.408	14.595	< 0.001
Age of the child	0.065	− 0.223	0.710	0.306
Sex of the child = boy	− 0.019	− 0.510	0.340	0.694
School year of the child	− 0.142	− 0.197	− 0.007	0.035*
Ability to express feelings	− 0.129	− 1.234	− 0.031	0.039*
Psychological Well-Being score	0.081	− 0.016	0.638	0.062
Diabetes Disease Knowledge score	0.105	0.052	0.147	< 0.001*
Knowledge of Diabetes Pharmacotherapy score	0.072	− 0.072	0.307	0.224
Recent serum glycated hemoglobin Hba1c level	− 0.108	− 0.179	− 0.010	0.028*

^*^Significance level at 0.05

## Discussion

The level of diabetes knowledge among the participating children was varied. Although a significant proportion of participants demonstrated correct knowledge regarding certain aspects of diabetes, such as the link between sugar consumption and diabetes, there were also areas of misconception, as indicated by the high percentage of incorrect answers. Insufficient knowledge about diabetes mellitus among patients has been observed in other studies [24–27]. Nonetheless, comparing our findings with those studies is challenging due to variations in the instruments used and the diverse ethnic and age groups involved in each study.

On the other hand, the results revealed a lack of knowledge about diabetes pharmacotherapy among the participants, with only 11.1% having some or complete information about their insulin. Poor knowledge is one of the primary factors contributing to inadequate self-care behaviors [28]. Given that T1DM often develops in young individuals who may have limited knowledge for effectively managing their condition [29], scholars emphasize the importance of continuous diabetes education for individuals living with diabetes which has been proven to enhance self-care practices, coping mechanisms, and lifestyle adjustments [24, 30, 31]. Consequently, effective self-management significantly reduces the likelihood of experiencing microvascular and macrovascular complications, as well as mortality [24]. The results of the present study indicate that a comprehensive understanding of the disease itself (*p* < 0.001), rather than of the insulin, is one of the determinants of effective self-management among the participants.

The findings provide insights into the psychological well-being of the children with diabetes. The mean scores suggest a mixed psychological profile, with moderate levels of happiness and feeling fine reported by the participants. However, it is concerning to note the considerable presence of negative emotional experiences, such as exhaustion, worry, and sadness, as reported by the children. The management of diabetes in younger individuals exposes them to higher risks of psychological distress, with adolescents being 2.3 times more likely to experience emotional and mental problems [32]. The considerable and ongoing efforts required for self-management, physical well-being, adherence to treatment, and social functioning can often lead to increased vulnerability to anxiety, depression, and other forms of emotional distress associated with diabetes [33]. While the study results showed a correlation between psychological well-being and self-management of diabetes mellitus (*r* = 0.112), psychological well-being was not found to be a significant determinant of the effectiveness of self-management among the participants (*p* = 0.062).

The children’s responses to the subscales of diabetic self-management in the present work revealed a range of scores across different aspects. The highest mean score was observed for the attention to readings, suggesting that children were relatively engaged in this aspect of self-care. However, the lowest self-rated diabetic self-care activity reported by the children was consistently carrying an item indicating their diabetic condition, such as a bracelet, indicating potential challenges or lower adherence to this specific self-care activity.

The results of the multivariate linear regression analysis provide valuable insights into the predictors of the children with diabetes self-management overall score. These findings suggest that factors such as glycemic control and knowledge about diabetes mellitus significantly influence overall self-management behaviors. It is worth noting that better glycemic control is not only influenced by self-care behaviors as reported by previous literature [34–36] but also has a reciprocal relationship with self-care management, i.e., improved glycemic control is associated with enhancing adherence to self-care activities in our study.

### Study limitations

Several limitations were present in our study. Firstly, the assessment of self-care practices relied on the participants’ self-reported responses, which is less reliable than direct observation or confirmation of their actual performance of these behaviors. Similarly, the evaluation of knowledge and psychological well-being also relied on self-reported measures, introducing the possibility of response bias and potential underestimation or overestimation of participants’ actual knowledge and psychological well-being. Additionally, as this is a hospital-based study using a convenience sample, the generalizability of the results may be affected.

## Conclusion

The findings of this study underscore the complexity of diabetic self-management in children and adolescents and the need for a multi-dimensional approach to diabetes management that encompasses not only medical interventions but also psychological and educational support. Implementing tailored education programs can play a significant role in improving the understanding of diabetes among these children, especially in areas where knowledge gaps are identified.

## Data Availability

The datasets used and analyzed during the current study are available from the corresponding author upon reasonable request.

## Abbreviations

DSCM: Diabetes self-care management
T1DM: Type 1 diabetes mellitus
DM: Diabetes mellitus
HbA1c: Glycated hemoglobin
SMOD-A: Self-Management of type 1 diabetes in Adolescents scale
ChilD-S: Children’s depression screener
DKQ: Diabetes knowledge questionnaire
KR-20: Kuder-Richardson’s
SD: Standard deviation
